# FNDC4 and FNDC5 Attenuate SARS‐CoV‐2 S1‐Induced Inflammatory Responses in Human Adipose Tissue

**DOI:** 10.1111/eci.70215

**Published:** 2026-04-28

**Authors:** Gabriela Neira, Julia Hernández‐Castañeda, Victoria Catalán, Sara Becerril, Marina Martín, Víctor Valentí, Rafael Moncada, Camilo Silva, Javier Gómez‐Ambrosi, Javier Escalada, Gema Frühbeck, Amaia Rodríguez

**Affiliations:** ^1^ Metabolic Research Laboratory Clínica Universidad de Navarra Pamplona Spain; ^2^ CIBER Fisiopatología de la Obesidad y Nutrición (CIBEROBN) Instituto de Salud Carlos III Madrid Spain; ^3^ Institute for Nutrition and Health University of Navarra Pamplona Spain; ^4^ Obesity and Adipobiology Group Instituto de Investigación Sanitaria de Navarra (IdiSNA) Pamplona Spain; ^5^ Department of Pathology, Anatomy and Physiology University of Navarra Pamplona Spain; ^6^ Department of Surgery Clínica Universidad de Navarra Pamplona Spain; ^7^ Department of Anesthesia Clínica Universidad de Navarra Pamplona Spain; ^8^ Department of Endocrinology and Nutrition Clínica Universidad de Navarra Pamplona Spain

**Keywords:** ACE2, adipose tissue inflammation, myokines, obesity, SARS‐CoV‐2

## Abstract

**Background:**

Adipose tissue is recognised as a SARS‐CoV‐2 reservoir and potential infection site. We herein characterised SARS‐CoV‐2 entry points in visceral (VAT) and subcutaneous (SAT) adipose tissue from people with obesity and determined whether the adipo‐myokines FNDC4 and FNDC5 modulate SARS‐CoV‐2 spike glycoprotein subunit 1 (S1)–induced inflammation in adipocytes and macrophages.

**Methods:**

Plasma concentrations of FNDC4, FNDC5 and angiotensin‐converting enzyme 2 (ACE2) were measured in 183 participants with obesity and normal weight. Expression of SARS‐CoV‐2 host cell entry receptors was analysed in paired VAT and SAT biopsies (*n* = 121). The effects of FNDC4 and FNDC5 on S1‐induced inflammatory responses were evaluated in vitro using human visceral adipocytes and THP‐1‐derived macrophages.

**Results:**

Obesity was associated with higher circulating ACE2 and increased expression of SARS‐CoV‐2 entry receptors (ACE2, CD147, DPP4 and neuropilin‐1) in VAT, whereas plasma FNDC4 and FNDC5 levels were reduced. FNDC4, FNDC5 and ACE2 co‐localised with macrophage populations in VAT, and *FNDC4* and *FNDC5* transcripts positively correlated with genes involved in viral entry and priming. Both adipo‐myokines attenuated S1‐induced M1 macrophage polarisation and HMGB1 secretion and reduced HMGB1 expression in adipocytes.

**Conclusion:**

Reduced FNDC4 and FNDC5 levels in obesity may amplify SARS‐CoV‐2 S1‐induced inflammatory responses in VAT macrophages and adipocytes.

## Graphical abstract

In obesity, reduced FNDC4 and FNDC5 amplify SARS‐CoV‐2 S1–driven inflammation in visceral adipose tissue. These adipo‐myokines limit adipocyte–macrophage inflammatory signalling, suggesting a protective role against viral‐induced adipose tissue dysfunction.

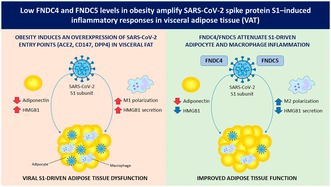

## 1 Introduction

Both sedentary lifestyle and obesity have been identified as significant risk factors for severe clinical outcomes, including invasive mechanical ventilation, acute respiratory distress syndrome, thrombosis and mortality, in patients with COVID‐19 admitted to the intensive care unit [1, 2, 3, 4]. Excessive adipose tissue accumulation may further contribute to increased viral spread, prolonged viral shedding, heightened immune activation and amplification of the cytokine response [5]. In this sense, adipose tissue serves not only as a reservoir for SARS‐CoV‐2 due to its expression of angiotensin‐converting enzyme 2 (ACE2) and other host cell receptors [5], but also as a direct target for viral infection, eliciting an inflammatory response through the infection of adipocytes and macrophages [6, 7]. SARS‐CoV‐2 infection impairs adipocyte phenotype and gene expression, leading to adipocyte dysfunction [7, 8]. In aged golden Syrian hamsters, viral infection reduces adipocyte size and prolongs the presence of large crown‐like structures (CLS) in subcutaneous fat, indicating impaired macrophage‐mediated clearance of dead adipocytes [9]. Increased adipose tissue browning and atrophy have been reported in SARS‐CoV‐2‐infected mouse and hamster models, as well as in *post‐mortem* adipose tissue from patients with COVID‐19 [10], which might explain the COVID‐19‐associated weight loss. In obesity, adipose tissue undergoes chronic low‐grade inflammation driven by the sustained recruitment and activation of pro‐inflammatory immune cells such as macrophages, T cells and neutrophils, particularly within visceral fat [11]. SARS‐CoV‐2 triggers both the innate and adaptive immune systems, with the spike glycoprotein subunit 1 (S1) playing a pivotal role in mediating viral entry via ACE2 binding [12]. Notably, the S1 subunit can trigger excessive immune activation, particularly in myeloid cells, thereby contributing to the development of the cytokine storm. Simultaneously, dysfunctional adipocytes worsen this pro‐inflammatory environment by secreting cytokines, chemokines and alarmins [11].

Regular physical activity is associated with decreased systemic inflammation and more favourable COVID‐19 outcomes among infected individuals [3], although the underlying molecular mechanisms remain incompletely understood. Growing evidence points to the role of factors released by contracting muscle, termed myokines, in mediating the exercise benefits across various diseases, including obesity and COVID‐19 [13]. Notably, many myokines are also secreted by adipocytes and are referred to as adipo‐myokines [14]. Among them, special attention has been paid to irisin, the C‐terminally cleaved portion of fibronectin type III domain containing 5 (FNDC5) protein, which increases in circulation in response to exercise [15]. FNDC5/irisin binds αV integrins on adipocytes promoting fat browning and inhibiting adipogenesis [15, 16, 17, 18, 19]. FNDC4, a homologue of FNDC5, shares similar anti‐obesity actions via binding to its putative receptor G‐protein coupled receptor 116 (GPR116) in human visceral adipocytes [16]. In addition to their metabolic effects, both FNDC4 and FNDC5 exhibit anti‐inflammatory properties, counteracting adipose tissue inflammation by mitigating macrophage M1 polarisation, phagocytic activity and pro‐inflammatory cytokine release [20, 21, 22, 23, 24]. This dual action highlights their potential as regulators of both metabolic and immune homeostasis. Notably, lower serum irisin concentrations at hospital admission in people with COVID‐19 were inversely associated with disease severity and poorer prognosis, implicating irisin in the pathophysiology of COVID‐19 [25]. Accordingly, FNDC5/irisin has emerged as a potential therapeutic target, given its putative ability to attenuate the cytokine storm and lessen disease severity [26, 27]. In this context, FNDC4 and FNDC5 downregulate SARS‐CoV‐2 entry and infection genes in human visceral adipocytes [28], with FNDC5 exerting similar effects in subcutaneous adipocytes [29]. Consistently, FNDC5 prevents viral particle uptake and cytotoxicity in subcutaneous adipocytes exposed to a SARS‐CoV‐2 pseudovirus [30]. Moreover, FNDC4 and FNDC5 attenuate SARS‐CoV‐2 spike protein S1‐induced pyroptosis, apoptosis and necroptosis in human visceral adipocytes [28].

In COVID‐19 autopsy cases, detection of SARS‐CoV‐2 RNA in adipocytes was accompanied by inflammatory infiltrates [6], suggesting a potential adipocyte‐macrophage crosstalk during viral infection. Based on this, we hypothesised that FNDC5 and its orthologue FNDC4 play a role in the immune dialogue between adipocytes and macrophages in the setting of the SARS‐CoV‐2 infection. Accordingly, our objectives were to: (1) investigate differences in circulating ACE2, FNDC4 and FNDC5 levels in people with obesity stratified by insulin resistance status; (2) characterise ex vivo the association of FNDC4 and FNDC5 with the expression of SARS‐CoV‐2 entry receptors and processing enzymes in human visceral (VAT) and subcutaneous (SAT) adipose tissue; (3) evaluate in vitro the effects of FNDC4 and FNDC5 on SARS‐CoV‐2 S1‐induced macrophage polarisation and adipocyte dysfunction; and (4) explore adipocyte‐macrophage crosstalk by reproducing experiments in macrophages in the presence of adipocyte‐conditioned media.

## 2 Material and Methods

### 2.1 Participants and Experimental Design

In this case–control study, plasma levels of FNDC4 and FNDC5 were measured in 183 participants with normal weight (BMI < 25 kg/m^2^) undergoing Nissen fundoplication (*n* = 34) and with severe obesity (BMI ≥ 40 kg/m^2^) submitted to bariatric surgery (sleeve gastrectomy or Roux‐en‐Y gastric bypass) (*n* = 149). Body fat (BF) was estimated by air‐displacement plethysmography (Bod‐Pod; COSMED, Rome, Italy). Visceral adiposity was assessed by abdominal bioelectrical impedance with the ViScan device (Tanita AB‐140, Tanita Corp., Tokyo, Japan). Physical activity level (PAL) was assessed using a validated questionnaire [31]. A 2‐h 75‐g oral glucose tolerance test (OGTT) was performed in patients with severe obesity for classification as normoglycaemia (NG), impaired glucose tolerance (IGT), or type 2 diabetes (T2D), according to the criteria of the Expert Committee on the Diagnosis and Classification of Diabetes [32]. Inclusion criteria comprised age ≥ 18 years, absence of psychiatric disorders, and availability of clinical, anthropometric and biochemical data. Exclusion criteria were liver and renal diseases not related to obesity, infectious diseases, cancer, pregnancy, or lactation. The study protocol adhered to the Declaration of Helsinki and was approved by the Ethical Committee responsible for the research (ref. 2020.187). All participants provided written informed consent.

### 2.2 Blood Assays

After an overnight fast, venous blood samples were collected and centrifuged at 3000×*g* for 15 min at 4°C for serum and plasma separation. Biochemical measurements were performed as earlier described [16]. Adipocyte insulin resistance was estimated using the Adipo‐IR index, calculated as fasting free fatty acids (FFA) (mmol/L) × fasting insulin (pmol/L). Plasma FNDC4 (MBS9332722, MyBiosource, San Diego, CA), FNDC5 (SEN576Hu, USCN Life Science Inc., Wuhan, China) and ACE2 (RAG006R, BioVendor, Brno, Czech Republic) were measured by ELISA. Intra‐ and inter‐assay coefficients of variation for all assays were < 15%.

### 2.3 Adipose Tissue Handling

Paired VAT and SAT samples (*n* = 121) were collected during surgery from patients with normal weight (Nissen fundoplication) and obesity (sleeve gastrectomy or Roux‐en‐Y gastric bypass). Fat samples were immediately stored at −80°C for gene expression analyses. A portion of VAT was fixed in 4% formaldehyde for histological studies. Another portion of VAT was used to isolate adipocytes and stromal vascular fraction cells (SVFC) via 2% collagenase digestion [33]. Total RNA was isolated and purified using QIAzol Reagent (Qiagen, Hilden, Germany) and the RNeasy Lipid Tissue Mini Kit (Qiagen) for AT and adipocytes and TRIzol Reagent (Invitrogen, Paisley, UK) and RNeasy Mini Kit (Qiagen) for SVFC, according to the manufacturer's protocols.

### 2.4 Adipocyte and Macrophage Culture and Treatment

Human SVFC were seeded at a density of 2 × 10^5^ per well in six‐well plates and cultured to confluence using Dulbecco's Modified Eagle Medium/F‐12 (DMEM/F‐12) (Invitrogen) and then treated for 10 days with a specific cocktail of hormones for adipocyte differentiation, as previously described [33]. Human THP‐1 leukaemic monocytes (Sigma, St. Louis, MO, USA) were seeded at a density of 3 × 10^5^ per well in six‐well plates and cultured for 24 h in RPMI Medium 1640 ATCC (A10491‐01, Invitrogen) supplemented with 10% fetal bovine serum (FBS) and 20 ng/mL phorbol 12‐myristate 13‐acetate (PMA) (P8139, Sigma) for macrophage differentiation. Afterwards, THP‐1 macrophages were cultured for 72 h with RPMI supplemented with 10% FBS.

Human differentiated adipocytes and THP‐1 macrophages were cultured in serum‐free medium for 24 h, and then treated with SARS‐CoV‐2 S1 protein (10 ng/mL) (ab273068, abcam Ltd., Cambridge, UK) in the presence or absence of FNDC4 (10 ng/mL) or FNDC5 (10 ng/mL) (AG‐40B‐0124‐C010 and AG‐40B‐0102‐C010, respectively, Adipogen, Liestal, Switzerland) for 24 h. Macrophage‐conditioned media (MCM) were collected following treatment with different stimuli, centrifuged at 1000×*g* for 10 min and stored at −80°C for subsequent studies. In a second set of experiments, macrophages were exposed to adipocyte‐conditioned media (ACM) diluted to 40%. The concentrations of FNDC4, FNDC5 and SARS‐CoV‐2 S1 were chosen based on prior experiments [16, 28].

### 2.5 Adipocyte *FNDC4* and *FNDC5* Gene Knockdown by esiRNA Transfection

MISSION esiRNA targeting human *FNDC4* (EHU045461) or *FNDC5* (EHU071871) mRNA and MISSION siRNA Universal Negative Control number 1 (SIC001) were obtained from Sigma‐Aldrich. Control, *FNDC4* and *FNDC5* esiRNAs (final concentration: 100 pmol/L) were complexed with 5 μL of Lipofectamine 2000 reagent (Invitrogen) in serum‐free Opti‐MEM I (Invitrogen), according to the manufacturer's protocol. After incubation for 20 min at room temperature (RT), complexes were added to cells and incubated at 37°C for 4 h. Transfection medium was removed and replaced with fresh adipocyte culture media. Knockdown efficiency was assessed 24 h post‐transfection by real‐time PCR. Treatment with MISSION esiRNA *FNDC4* or *FNDC5* treatment resulted in reductions of 86% (*p* < 0.01) and 66% (*p* < 0.05) of *FNDC4* and *FNDC5* mRNA levels, respectively (Figure S1).

### 2.6 Detection of HMGB1 in Macrophage Culture Media

The concentrations of HMGB1 (NBP2‐62766, Novus Biologicals, Cambridge, UK) released to the MCM were assessed by commercially available ELISA kits according to the manufacturer's protocols. The intra‐ and inter‐assay coefficients of variation were < 4.5% and 5.1%, respectively.

### 2.7 Real‐Time PCR

Transcript levels of genes encoding for SARS‐CoV‐2 entry and processing factors (*ACE2*, *CD147*, *DPP4, FURIN*, *NRP1*), adipo‐myokines (*FNDC4* and *FNDC5*) as well as macrophage polarisation (*NOS2, IL1B, ARG1, IL4, HMGB1*) were quantified by real‐time PCR (7300 Real‐Time PCR System; Applied Biosystems, Foster City, CA, USA). Primers and probes (Table S1) were designed using the software Primer Express 2.0 (Applied Biosystems) and purchased from Genosys (Sigma). All results were normalised for *18S* rRNA expression (Applied Biosystems), and relative quantification was calculated using the 2^−∆∆Ct^ formula [34].

### 2.8 Western‐Blot Studies

Protein samples (20 μg) were separated by SDS‐PAGE (Mini‐PROTEAN TGX Precast Gels, Bio‐Rad Laboratories Inc., Hercules, CA, USA) under denaturing conditions and transferred to a nitrocellulose membrane (Bio‐Rad) for immunoblotting. Blots were incubated overnight at 4°C with rabbit polyclonal anti‐adiponectin (Acrp30) (sc‐17044, Santa Cruz Biotechnology, Santa Cruz, CA, USA), anti‐HMGB1 (NB100‐2322, Novus Biologicals) or mouse monoclonal anti‐β‐actin (A5441, Sigma) antibodies diluted 1:1000 in Tris‐buffered saline with Tween 20 (TBS‐T) containing 5% non‐fat dry milk. The antigen–antibody complexes were visualised using HRP‐conjugated secondary antibodies (1:5000) and the Pierce ECL Plus Western‐blotting Substrate (Thermo Fisher Scientific, Waltham, MA, USA). The intensity of the bands was determined using the Gel Doc gel documentation system and Quantity One 4.5.0 software (Bio‐Rad) and normalised with β‐actin density values.

### 2.9 Immunohistochemistry and Double Immunofluorescence Studies

Sections of formalin‐fixed, paraffin‐embedded VAT and SAT (6 μm) were used to detect CD68, ACE2, FNDC4 and FNDC5 by immunohistochemistry employing the indirect immunoperoxidase method [16]. Slides were incubated overnight at 4°C with mouse monoclonal anti‐CD68 (ab31630, abcam, Cambridge, UK), mouse monoclonal anti‐ACE2 (MAB9331, R&D Systems, Minneapolis, MN, USA), rabbit polyclonal anti‐FNDC4 (SAB1401807, Sigma) and rabbit polyclonal anti‐FNDC5 (ab93373, Abcam) diluted 1:100 in TBS. After three washes in TBS, slides were incubated with pure DAKO Real EnVision anti‐rabbit/mouse HRP polymer (K5007; Dako, Golstrup, Denmark) for 1 h at RT. The peroxidase reaction was visualised using a 0.5 mg/mL diaminobenzidine (DAB)/0.03% H_2_O_2_ solution diluted in 50 mmol/L Tris–HCl, pH 7.36. Slides were counterstained with Harris haematoxylin solution (Sigma). Negative control slides without primary antibody were included to assess non‐specific staining.

For double immunofluorescence studies, VAT and SAT sections were incubated overnight at 4°C with the above‐mentioned primary antibodies against ACE2, FNDC4 and FNDC5 together with the pan‐macrophage marker CD68 antibody (all in dilution 1:100 in TBS‐T). Following three washes, sections were incubated with Alexa Fluor 488‐conjugated goat anti‐mouse IgG (A32723TR, Invitrogen), Alexa Fluor 568 goat anti‐mouse IgG (A11004, Invitrogen), or Alexa Fluor 594 goat anti‐rabbit IgG (A11012, Invitrogen) diluted 1:200 in TBS‐T for 2 h at RT. After washing, coverslips were mounted using Vectashield with DAPI (H‐1500‐10, Vector Laboratories, Burlingame, CA, USA) for nuclear counterstaining. Fluorescent images were acquired using an Axiophot fluorescence microscope (Zeiss, Göttingen, Germany) equipped with pE‐300 lite (CoolLED) LED light source and a Digital Sight DS‐5MC camera (Nikon), operated with NIS‐Elements software.

### 2.10 Statistical Analysis

Statistical analyses were conducted using the SPSS 15.0 software. Data are expressed as mean ± standard deviation (SD) or standard error of the mean (SEM), as indicated. Differences between mean values were determined using Student's *t*‐test, one‐way or two‐way ANOVA followed by Scheffé's or Dunnett's tests, where appropriate, for quantitative variables and assessment of *χ* ^2^ distributions for categorical variables. Differences between groups adjusted for age and PAL were explored by ANCOVA. Potential outliers were identified by the Grubbs' test. Stepwise multiple linear regression analysis was used to analyse the association between variables. A *p* value < 0.05 was considered statistically significant.

## 3 Results

### 3.1 Increased Expression of SARS‐CoV‐2 Entry Factors in Visceral and Subcutaneous Adipose Tissue From Patients With Severe Obesity

**FIGURE 1:**
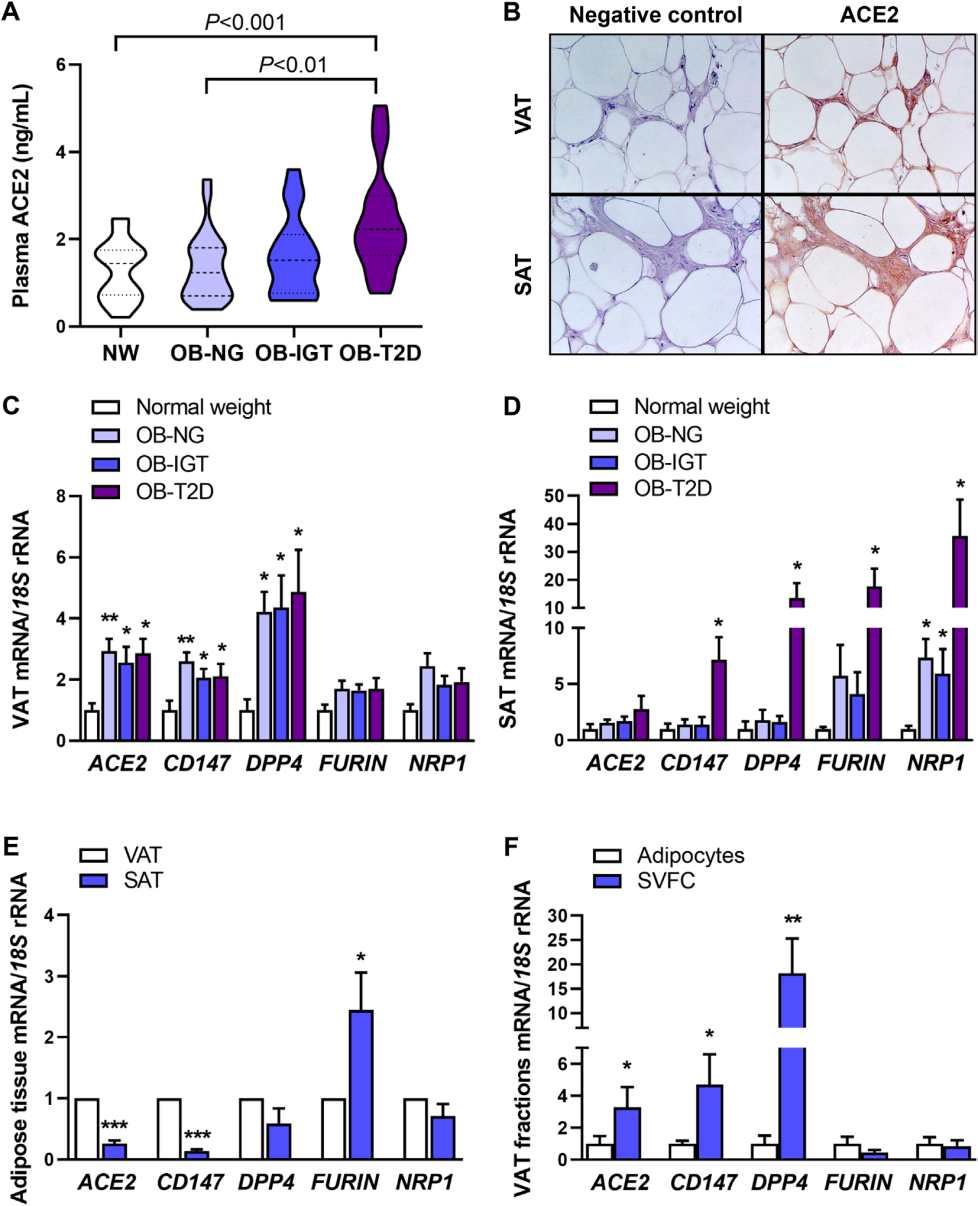
Increased expression of ACE2 and alternative SARS‐CoV‐2 cell entry points in adipose tissue from patients with obesity and insulin resistance. (A) Fasting plasma ACE2 levels in normal‐weight individuals and patients with obesity with normoglycemia (NG), impaired glucose tolerance (IGT) or type 2 diabetes (T2D). (B) Immunohistochemical detection of ACE2 (*right panels*) in visceral (VAT, *upper panels*) and subcutaneous (SAT, *bottom panels*) adipose tissue (magnification ×200) (*n* = 6). No immunoreactivity was found without primary antibody (negative control. *left panels*). Gene expression of SARS‐CoV‐2 host cell receptors (*ACE2. CD147. DPP4* and *NRP1*) and the enzyme responsible for SARS‐CoV‐2 S1/S2 protein priming (*FURIN*) in paired VAT (C) and SAT (D) biopsies. Comparison of SARS‐CoV‐2 host cell receptor expression (E) between VAT and SAT and in (F) VAT adipocyte and stroma‐vascular (SVFC) subfractions obtained from patients with obesity. The gene expression in normal‐weight patients, VAT or adipocyte fractions were considered 1. Statistical differences were assessed by one‐way ANOVA followed by Tukey's test or by two‐tailed paired Student's *t*‐test, where appropriate. *p < 0.05, **p < 0.01, ***p < 0.001 versus normal‐weight controls or adipocyte fractions.

The clinical characteristics of the cohort are presented in Table 1. Circulating levels of ACE2, the primary receptor for SARS‐CoV‐2, were analysed for associations with established determinants of severe COVID‐19, including age, male sex, obesity and T2D [35]. In an analysis of covariance (ANCOVA) adjusted for age, plasma ACE2 levels were increased in people with obesity (normal weight 1.27 ± 0.67 vs. obesity 1.74 ± 1.05 ng/mL, *p* < 0.05) and elevated in males (males 1.88 ± 0.93 vs. females 1.50 ± 1.00 ng/mL, *p* < 0.05). However, after additional adjustment for PAL, sex‐related differences in plasma ACE2 concentrations were no longer statistically significant (*p* = 0.179), reinforcing the influence of fitness on circulating ACE2 levels [36]. When participants were stratified by insulin sensitivity, elevated ACE2 concentrations were observed exclusively in individuals with obesity and insulin resistance (*p* < 0.01), but not in those with obesity and NG or in the healthy‐weight group (Figure 1A). Consistently, plasma ACE2 positively correlated with fasting glycaemia (*r* = 0.37, *p* = 0.003) and with 2‐h glucose levels after the OGTT (*r* = 0.43, *p* = 0.003).

**TABLE 1:** Clinical characteristics of the participants of the study.

	Normal weight NG	Obesity NG	Obesity IGT	Obesity T2D	*p*
*n*	34	66	43	40	—
Sex (men/women)	16/18	22/44	15/28	21/19	0.174
Age (years)	47 ± 16	39 ± 12^a^	44 ± 10	46 ± 9	**0.004**
Body weight (kg)	64 ± 14	126 ± 30^a^	127 ± 22^a^	131 ± 26^a^	**< 0.00001**
BMI (kg/m^2^)	22.8 ± 3.3	45.8 ± 9.9^a^	45.4 ± 6.6^a^	47.2 ± 8.9^a^	**< 0.00001**
Body fat (%)	20.9 ± 6.7	51.8 ± 6.8^a^	53.6 ± 5.9^a^	50.1 ± 7.1^a^	**< 0.00001**
Fat free mass (%)	79.1 ± 6.9	48.2 ± 6.7^a^	46.4 ± 5.8^a^	49.9 ± 7.1^a^	**< 0.00001**
Visceral fat (%)	14.7 ± 4.4	54.2 ± 5.4^a^	55.7 ± 6.4^a^	51.6 ± 5.4^a^	**< 0.00001**
Waist circumference (cm)	75 ± 12	127 ± 20^a^	128 ± 16^a^	134 ± 16^a^	**< 0.00001**
PAL (A.U.)	1.92 ± 0.09	1.58 ± 0.11^a^	1.56 ± 0.11^a^	1.54 ± 0.10ª	**< 0.00001**
Glucose (mg/dL)	89 ± 12	90 ± 8	105 ± 10^b^	139 ± 49^a^, ^b^	**< 0.00001**
Glucose 2‐h OGTT (mg/dL)	—	119 ± 30	153 ± 31^b^	239 ± 79^b^	**< 0.00001**
Insulin (μU/mL)	5.7 ± 3.0	19.6 ± 13.1	17.6 ± 9.6	34.3 ± 33.4^a^, ^b^	**< 0.00001**
Insulin 2 h OGTT (μU/mL)	—	92.3 ± 8.0	146.5 ± 11.6	143.3 ± 20	**< 0.00001**
HOMA	1.2 ± 0.7	4.4 ± 3.2	4.7 ± 2.8	11.6 ± 13.3^a^, ^b^	**< 0.00001**
QUICKI	0.38 ± 0.04	0.32 ± 0.04^a^	0.31 ± 0.03^a^	0.30 ± 0.04^a^, ^b^	**< 0.00001**
FFA (mmol/L)	14.4 ± 6.2	21.1 ± 10.1	22.8 ± 13.2^a^	25.7 ± 14.2^a^	**0.003**
Glycerol (mg/dL)	18.6 ± 11.4	24.7 ± 18.9	25.8 ± 19.1	34.2 ± 25.1^a^	**0.031**
Adipo‐IR index	19 ± 8	99 ± 74	102 ± 86	191 ± 162^a^, ^b^	**< 0.00001**
Triacylglicerol (mg/dL)	70 ± 28	103 ± 45	130 ± 97	172 ± 136^a^, ^b^	**< 0.003**
Total cholesterol (mg/dL)	188 ± 29	190 ± 33	199 ± 36	185 ± 36	0.373
LDL‐cholesterol (mg/dL)	114 ± 20	117 ± 30	125 ± 32	115 ± 33	0.528
HDL‐cholesterol (mg/dL)	59 ± 14	52 ± 20	48 ± 11	40 ± 9^a^, ^b^	**< 0.00001**
CRP (mg/L)	2.1 ± 1.4	9.7 ± 12.1^a^	11.5 ± 14.0^a^	8.3 ± 7.4^a^	**0.002**
TNF‐α (ng/mL)	0.99 ± 0.49	1.43 ± 0.58^a^	1.99 ± 3.38^a^	1.88 ± 0.71^a^, ^b^	**0.001**
Uric acid (mg/dL)	4.2 ± 0.9	5.3 ± 1.2	5.9 ± 1.4^a^	6.2 ± 1.2^a^, ^b^	**< 0.00001**
Leptin (ng/mL)	6.7 ± 3.1	52.0 ± 28.1^a^	53.0 ± 25.0^a^	51.2 ± 34.3^a^	**< 0.00001**
AST (IU/L)	14 ± 5	15 ± 7	16 ± 5	18 ± 8	0.157
ALT (IU/L)	14 ± 11	19 ± 14	25 ± 12	29 ± 16^a^, ^b^	**0.002**
Alkaline phosphatase (IU/L)	104 ± 37	68 ± 37	85 ± 43	81 ± 36	**0.036**
γ‐GT (IU/L)	10 ± 4	23 ± 22	25 ± 15	31 ± 27^a^	**0.036**
Anti‐hypertensive therapy. *n* (%)	0 (0%)	16 (24.2%)^a^	10 (23.3%)^a^	21 (53.8%)^a^, ^b^	**< 0.00001**
Anti‐diabetic therapy, *n* (%)	0 (0%)	1 (1.5%)	1 (2.3%)	19 (48.7%)^a^, ^b^	**< 0.00001**
Lipid lowering therapy, *n* (%)	0 (0%)	6 (9.1%)	7 (16.3%)	18 (25.6%)^a^, ^b^	**0.017**

*Note:* Data are the mean ± standard deviation (SD). Differences between groups were analysed by one‐way ANOVA followed by Scheffé's test. Student's *t*‐test or χ^2^ test. where appropriate. Bold values denote statistically significant *p* values.

^a^ *p* < 0.05 versus patients with normal weight and NG.

^b^ *p* < 0.05 versus patients with obesity and NG.

Participants with severe obesity and insulin resistance showed significantly elevated (*p* < 0.001) circulating levels of ACE2 (Figure 1A). Immunohistochemical analysis confirmed ACE2 expression in both VAT and SAT (Figure 1B). In VAT, the expression of all key SARS‐CoV‐2 entry and processing factors, including *ACE2, CD147, DPP4* and *NRP1*, was significantly upregulated (all *p* < 0.05) in obesity (Figure 1C). In contrast, in SAT, these factors involved in SARS‐CoV‐2 infectivity (*CD147, DPP4, FURIN* and *NRP1*) were only upregulated (all *p* < 0.05) in people with obesity and T2D (Figure 1D). Depot‐specific comparisons revealed that *ACE2*, *CD147* and *DPP4* levels were significantly higher in VAT compared to SAT (Figure 1E). When assessing adipose tissue cellular fractions, *ACE2, CD147* and *DPP4* were more highly expressed (all *p* < 0.05) in SVFC than in mature adipocytes, while *FURIN* and *NRP1* showed no significant differences between the two fractions (Figure 1F).

### 3.2 Positive Association of FNDC4 and FNDC5 With SARS‐CoV‐2 Entry Factors in Adipose Tissue From Patients With Severe Obesity

**FIGURE 2:**
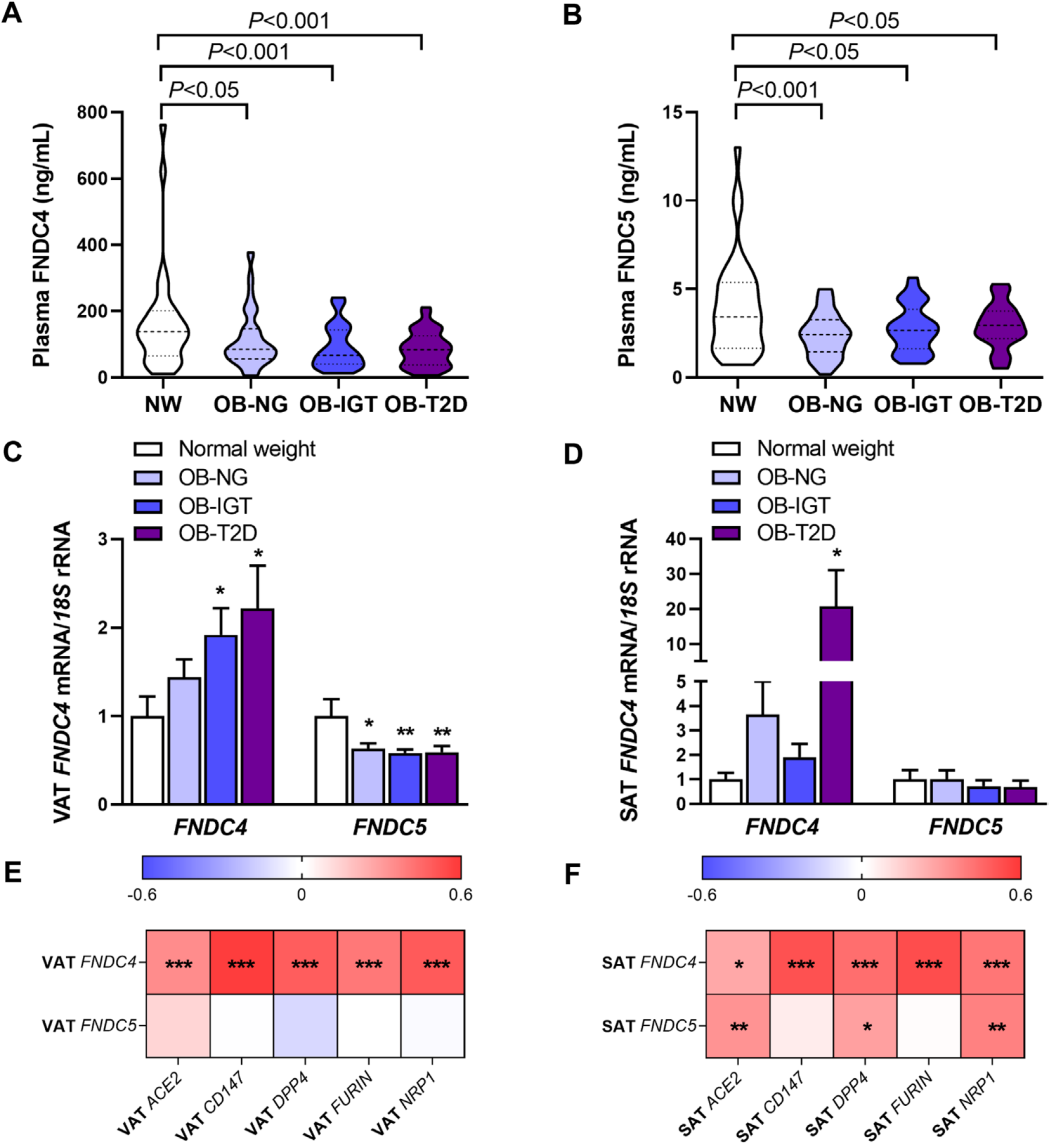
Association of visceral adipose tissue FNDC4 and FNDC5 expression with SARS‐CoV‐2 host cell receptors. Fasting plasma FNDC4 (A) and FNDC5 (B) in normal‐weight individuals and patients with obesity with normoglycemia (NG), impaired glucose tolerance (IGT), or type 2 diabetes (T2D). Transcript levels of *FNDC4* and *FNDC5* in visceral (VAT) (C) and subcutaneous (SAT) (D) adipose tissue biopsies. Heatmaps showing the correlation between the expression of *FNDC4* and *FNDC5* with that of SARS‐CoV‐2 host cell receptors in VAT (E) and SAT (F). Statistical differences were assessed by one‐way ANOVA followed by Tukey's test. *p < 0.05, **p < 0.01 and ***p < 0.001 versus normal‐weight controls.

Plasma FNDC4 concentrations varied between 5.3 and 1933.5 ng/mL (mean 131.5 ng/mL), while plasma FNDC5 levels ranged from 0.1 to 13.0 ng/mL (mean 2.9 ng/mL). Since physical activity is a major determinant of circulating FNDC5/irisin [15], its potential association with FNDC4 and FNDC5 levels was assessed. People living with obesity exhibited reduced physical activity regardless of insulin resistance status, as evidenced by significantly lower (*p* < 0.0001) values of PAL than participants with healthy weight (Table 1). Obesity was associated with reduced plasma concentrations of adipo‐myokines FNDC4 and FNDC5 (both *p* < 0.001), independently of insulin resistance status (Figure 2A,B). Adjustment for PAL resulted in the loss of group differences in circulating FNDC5 concentrations (*p* = 0.336), whereas differences in FNDC4 levels remained statistically significant (*p* < 0.05), suggesting differential regulation of these adipo‐myokines in response to physical activity [20, 37]. No sex‐related differences were observed in circulating levels of either molecule. Furthermore, univariate analyses revealed that PAL values were strongly correlated with fat‐free mass percentage (FFM%) (*r* = 0.69, *p* < 0.001), and moderately correlated with circulating concentrations of FNDC4 (*r* = 0.27, *p* < 0.05) and FNDC5 (*r* = 0.22, *p* < 0.05). In adipose tissue, expression patterns of FNDC4 and FNDC5 were differentially modulated by obesity and metabolic status (Figure 2C,D). FNDC4 expression was significantly upregulated in both VAT and SAT from people with obesity and T2D (both *p* < 0.05). In contrast, *FNDC5* expression was consistently downregulated (*p* < 0.05) in VAT across all obesity groups, regardless of insulin resistance status. Consistent with the observations in circulating adipo‐myokines, differences in VAT expression of *FNDC5*, but not *FNDC4*, were also no longer statistically significant after adjustment for PAL (*p* = 0.726 and *p* < 0.05). Interestingly, *FNDC4*, but not *FNDC5*, expression in VAT was positively associated with *ACE2* transcript levels (Figure 2E). Additionally, *FNDC4* mRNA expression in both VAT and SAT showed significant positive correlations with multiple SARS‐CoV‐2 entry and processing factors, including *CD147*, *DPP4*, *FURIN* and *NRP1* (Figure 2E,F). In SAT, *FNDC5* transcripts were also positively associated with *ACE2*, *DPP4* and *NRP1* mRNA levels.

**FIGURE 3:**
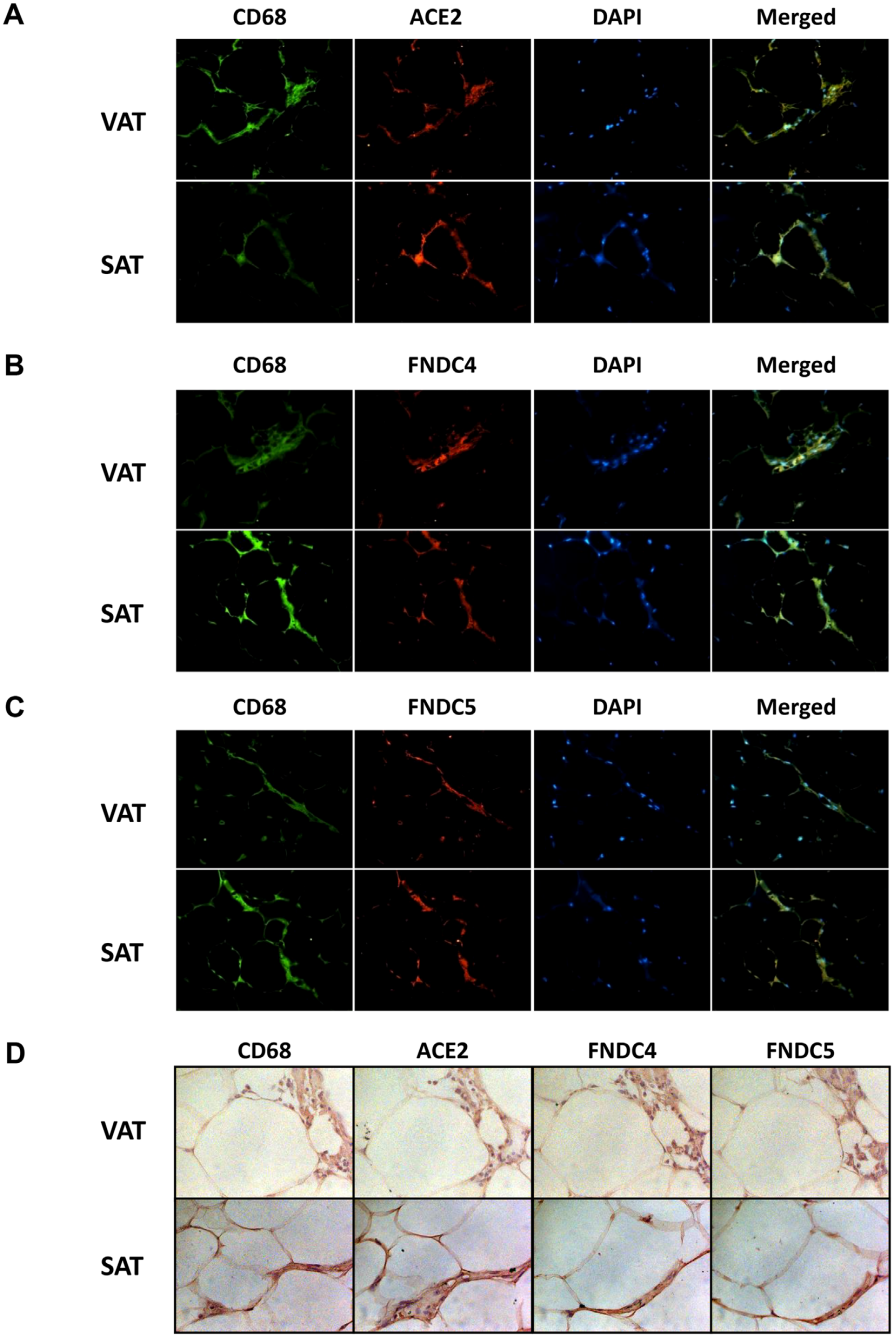
Co‐localisation of FNDC4 and FNDC5 with ACE2‐expressing macrophage populations in adipose tissue. Double immunofluorescence showing co‐localisation of pan‐macrophage marker CD68 (*green*) with (A) ACE2, (B) FNDC4 or (C) FNDC5 (*red*) in sections of visceral (VAT) (C) and subcutaneous (SAT) adipose tissue; nuclei were stained with DAPI (*blue*) (magnification 200×). Representative images of at least three separate experiments are shown. (D) Immunohistochemical staining for CD68, ACE2, FNDC4 and FNDC5 in paired VAT (G) and SAT (H) serial sections (magnification ×200) (*n* = 6).

Given the central role of macrophages in obesity‐associated COVID‐19 complications, we next investigated whether ACE2, FNDC4 and FNDC5 localise to macrophage populations within adipose tissue. To this end, double immunofluorescence and immunohistochemistry were performed using the pan‐macrophage marker CD68 in combination with specific antibodies against each protein. ACE2 (Figure 3A), FNDC4 (Figure 3B) and FNDC5 (Figure 3C) signals co‐localised with CD68‐positive cells, indicating their expression in macrophage populations in both VAT and SAT. Consistently, immunohistochemical analysis of serial VAT and SAT sections demonstrated co‐localisation of ACE2, FNDC4 and FNDC5 within macrophages (Figure 3D). These findings suggest that FNDC4 and FNDC5 may influence SARS‐CoV‐2 entry pathways in adipose tissue through macrophage‐associated mechanisms.

### 3.3 Effect of FNDC4 and FNDC5 on SARS‐CoV‐2 S1–Mediated Macrophage Polarisation and Crosstalk With Adipocytes

**FIGURE 4:**
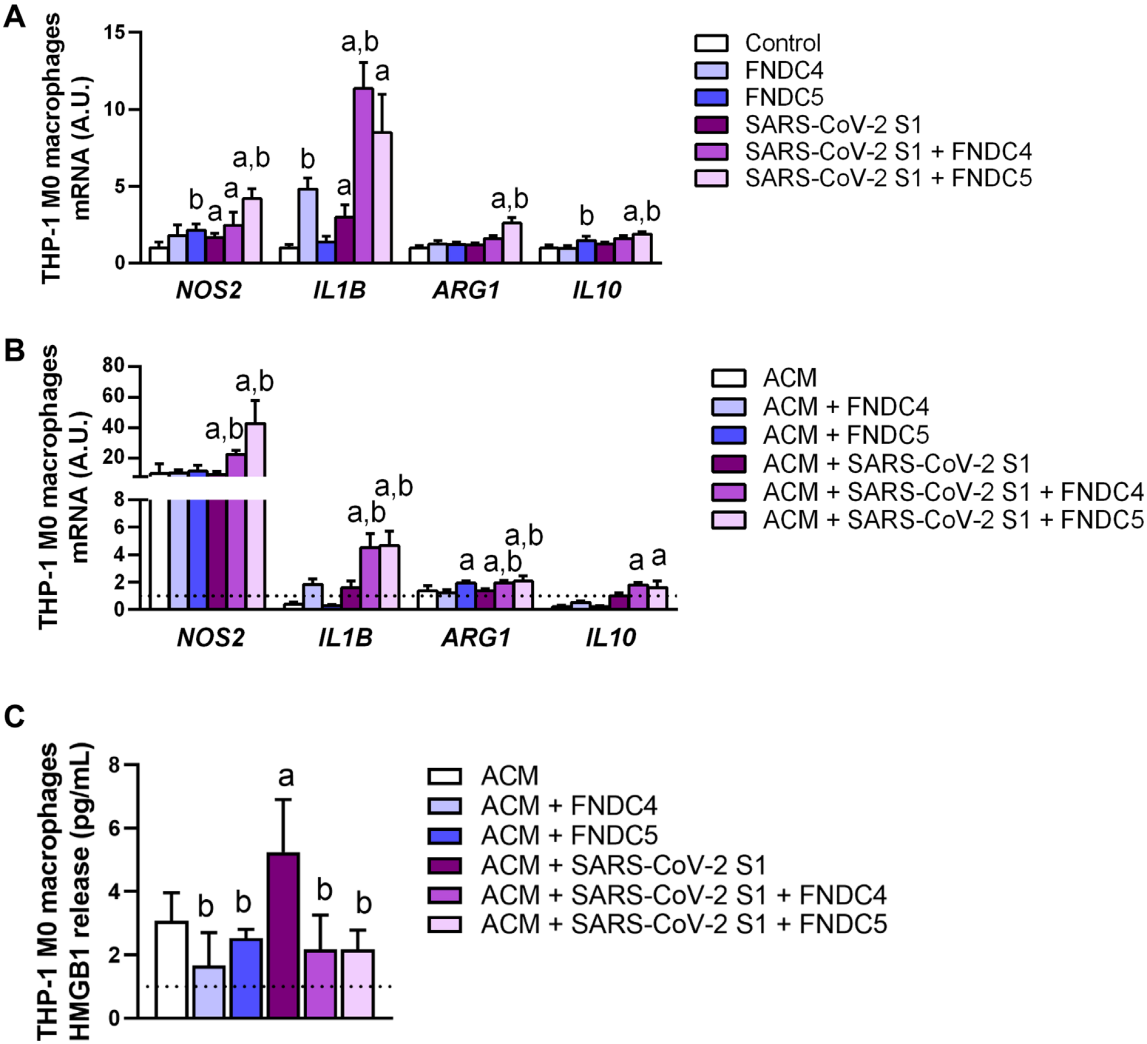
Effect of FNDC4 and FNDC5 on SARS‐CoV‐2 spike protein S1 subunit‐mediated macrophage M1 polarisation and crosstalk with adipocytes. Effect of co‐incubation of SARS‐CoV‐2 spike protein S1 subunit (100 ng/mL) with FNDC4 (10 ng/mL) or FNDC5 (10 ng/mL) on gene expression of several factors involved in the polarisation of human THP‐1 macrophages toward M1 (*NOS2*) or M2 (*ARG1*) phenotypes. as well as in the production of inflammatory (*IL1B*) and anti‐inflammatory (*IL10*) cytokines in the absence (A) or presence (B) of adipocyte‐conditioned medium (ACM 40%) derived from adipocyte cultures from patients with obesity (*n* = 10 per group). Gene expression in unstimulated THP‐1 M0 macrophages was assumed to be 1. (C) HMGB1 release to the culture media in macrophages exposed to ACM 40% in the presence of S1 subunit with FNDC4 or FNDC5. Statistical differences were analysed by two‐way ANOVA followed by a *post hoc* Tukey's test. ^a^ *p* < 0.05 effect of the SARS‐CoV‐2 S1 subunit; ^b^ *p* < 0.05 effect of treatment with FNDC4 or FNDC5.

The potential modulatory role of the adipo‐myokines FNDC4 and FNDC5 against the SARS‐CoV‐2 S1 inflammatory response was next investigated in macrophages. Human THP‐1‐derived macrophages were treated with S1 protein (100 ng/mL) for 24 h, a dose and duration previously shown to trigger pro‐inflammatory cytokine production [38]. S1 exposure upregulated (*p* < 0.05) the expression of *NOS2*, a marker of pro‐inflammatory M1 macrophages, as well as pro‐inflammatory cytokine *IL1B* (Figure 4A). Co‐incubation of S1 with FNDC4 (10 ng/mL) or FNDC5 (10 ng/mL) further enhanced *IL1B* transcription, suggesting an amplification of the inflammatory response. However, co‐incubation with FNDC5 (10 ng/mL) significantly upregulated (*p* < 0.05) in parallel *ARG1*, a marker of anti‐inflammatory M2 macrophages and the anti‐inflammatory cytokine *IL10*. These findings suggest that FNDC5, but not FNDC4, promotes an anti‐inflammatory macrophage phenotype and may mitigate S1‐induced pro‐inflammatory activation.

To further explore macrophage–adipocyte crosstalk in the context of viral challenge, THP‐1‐derived macrophages were co‐stimulated with S1 (100 ng/mL) and either FNDC4 or FNDC5 (10 ng/mL) in the presence of adipocyte‐conditioned medium (ACM, 40%) derived from adipocyte cultures from patients with obesity. As expected, treatment with ACM alone induced macrophage polarisation toward a pro‐inflammatory M1 phenotype, as evidenced by increased NOS2 expression (control 1.00 ± 0.37 vs. ACM 9.86 ± 6.52 A.U., *p* < 0.05) and decreasing transcription of the anti‐inflammatory cytokine *IL10* (control 1.00 ± 0.19 vs. ACM 0.20 ± 0.10 A.U., *p* = 0.012). In the presence of FNDC5 (10 ng/mL), *ARG1* expression was significantly upregulated (*p* = 0.017), supporting a shift toward an anti‐inflammatory M2 phenotype (*p* = 0.017) (Figure 4B). Although co‐incubation of S1 with FNDC4 or FNDC5 continued to promote expression of the pro‐inflammatory markers *NOS2* and *IL1B* (*p* < 0.05), a concurrent and significant induction of the anti‐inflammatory markers *ARG1* and *IL10* was observed in the presence of ACM. Moreover, macrophages exposed to ACM and S1 increased (*p* < 0.05) their secretion of HMGB1, an alarmin that initiates and maintains chronic adipose tissue inflammation [39, 40], with co‐treatment with FNDC4 and FNDC5 blunting (both *p* < 0.05) this increased release to the culture media (Figure 4C). These findings indicate that the adipocyte secretome from individuals with obesity amplifies the pro‐inflammatory activation of macrophages. However, both FNDC4 and FNDC5 appear to engage compensatory anti‐inflammatory pathways, suggesting a role in the negative regulation of inflammation under conditions of SARS‐CoV‐2 challenge and adipose tissue dysfunction.

### 3.4 The Pro‐Inflammatory Phenotype Induced by SARS‐CoV‐2 S1 Subunit in Adipocytes Is Ameliorated by FNDC4 and FNDC5 Treatment

**FIGURE 5:**
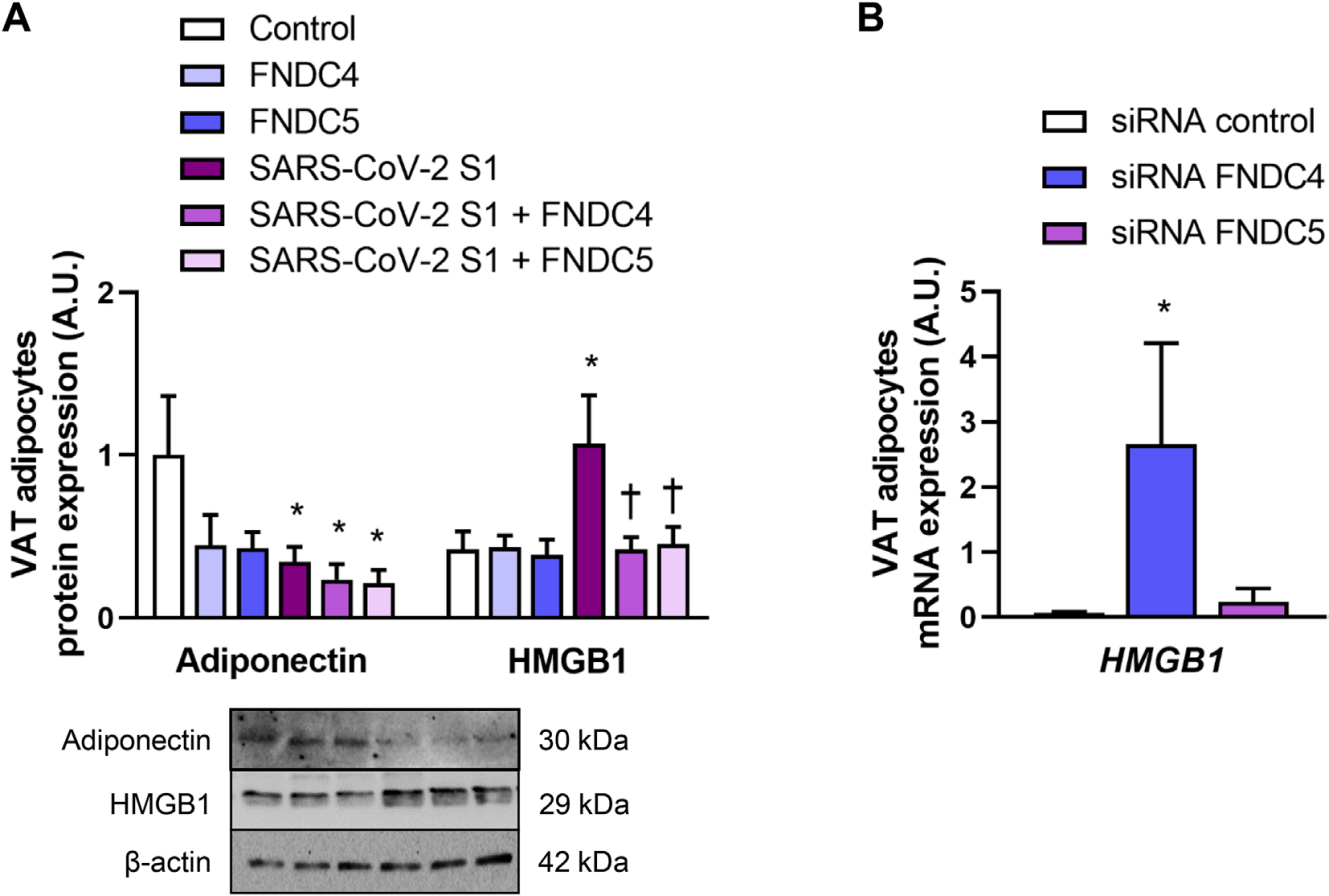
SARS‐CoV‐2 spike protein S1 subunit promotes a proinflammatory phenotype in visceral adipocytes. (A) Effect of co‐incubation of SARS‐CoV‐2 spike protein S1 subunit (100 ng/mL) with FNDC4 (10 ng/mL) or FNDC5 (10 ng/mL) on protein expression of adiponectin and HMGB1 expression in human visceral adipocytes (*n* = 6 per group). (B) *HMGB1* gene expression in *FNDC4*‐ and *FNDC5*‐knockdown adipocytes (*n* = 8 per group). Statistical differences were analysed by two‐way ANOVA or one‐way followed by a *post hoc* Tukey's test in case of interaction. *p < 0.05 versus unstimulated or siRNA control cells; †*p* < 0.05 versus SARS‐CoV‐2 S1‐treated cells.

We next examined whether exposure to SARS‐CoV‐2 S1 subunit induced inflammation‐related dysfunction in visceral adipocytes. Treatment with the S1 subunit markedly reduced the protein expression of the anti‐inflammatory adipokine adiponectin, with levels decreasing (*p* < 0.05) by approximately 70% compared with control adipocytes (Figure 5A). Concomitantly, S1 exposure significantly increased HMGB1 protein expression (Figure 5A). Co‐incubation with FNDC4 or FNDC5 markedly attenuated (*p* < 0.05) the S1‐induced increase in HMGB1 protein. Notably, gene silencing of *FNDC4*, but not *FNDC5*, significantly upregulated (*p* < 0.05) *HMGB1* transcript levels in human visceral adipocytes (Figure 5B), suggesting a distinct regulatory role for FNDC4 in modulating adipocyte inflammatory responses.

## 4 Discussion

Obesity is a major determinant for severe COVID‐19 and may contribute to SARS‐CoV‐2 infections occurring in fully vaccinated individuals [41, 42]. Adipose tissue is not only a viral reservoir [5], but also a direct target organ for SARS‐CoV‐2, with infection of adipocytes and macrophages triggering local and systemic inflammatory responses [6, 7]. Accordingly, excessive adipose tissue accumulation may facilitate more extensive viral dissemination, increased viral shedding, heightened immune activation and cytokine amplification [12]. The adipose tissue expresses SARS‐CoV‐2 host cell receptors ACE2, basigin‐1 or cluster of differentiation 147 (CD147) and dipeptidyl peptidase‐4 (DPP4) as well as spike glycoprotein (S)‐cleaving enzyme furin [11, 43, 44]. In addition, neuropilin‐1 (NRP1), a key SARS‐CoV‐2 co‐receptor that binds furin‐cleaved substrates and enhances viral entry and infectivity, is also expressed in adipose tissue [45]. We herein demonstrate that COVID‐19‐free individuals with obesity exhibit overexpression of several SARS‐CoV‐2 host cell receptors in VAT (ACE2, CD147 and DPP4), being primarily localised in SVFC, while expression in adipocytes of this depot is comparatively lower. SVFC play a central role in linking visceral obesity with chronic low‐grade inflammation (meta‐inflammation) and severe COVID‐19, as they release pro‐inflammatory cytokines such as TNF‐α, IL‐6 and IL‐1β, thereby creating a permissive inflammatory milieu that may exacerbate disease severity [46, 47]. In SAT, the upregulation of viral entry‐related factors (CD147, DPP4, furin and NRP1) was restricted to patients with obesity‐associated T2D. Consistent with previous reports, our findings support the concept that obesity enhances the expression of critical SARS‐CoV‐2 entry points, particularly ACE2 and neuropilin‐1 [45, 48]. Collectively, these data reinforce the notion that adipose tissue, and in particular VAT, serves as both a viral reservoir and a target organ, which is overactivated in obesity, potentially facilitating enhanced viral access and prolonged tissue persistence following infection.

**FIGURE 6:**
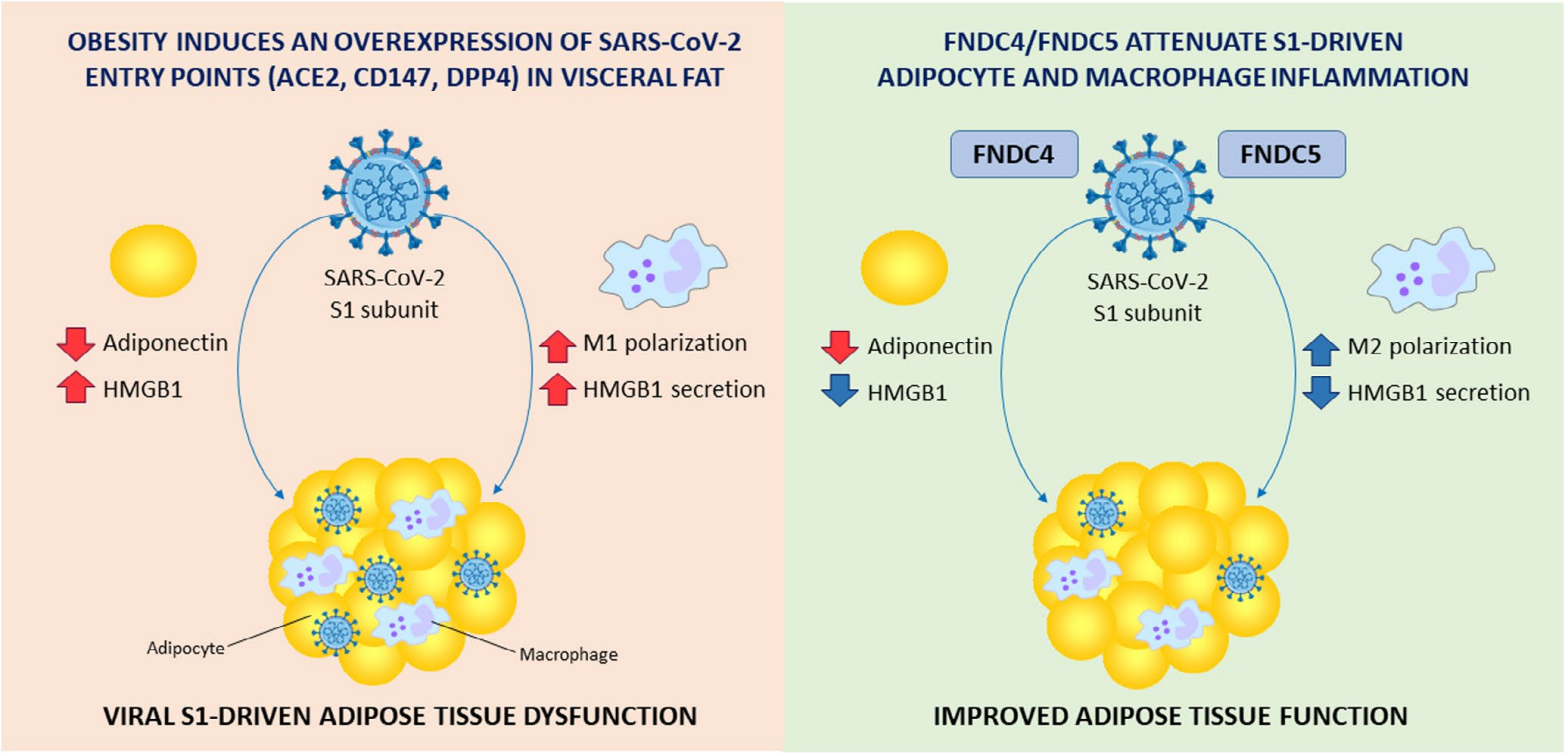
Schematic representation of the protective effects of FNDC4 and FNDC5 in attenuating SARS‐CoV‐2 spike glycoprotein S1–induced inflammatory responses in human adipose tissue.

Autopsy studies of patients with COVID‐19 have detected two principal cellular targets of SARS‐CoV‐2 infection within adipose tissue: adipocytes and a subpopulation of inflammatory adipose tissue‐resident macrophages [6]. During adipose tissue SARS‐CoV‐2 infection, adipocytes appear more permissive to viral replication, whereas infected resident macrophages primarily drive local and systemic inflammatory responses [6, 12]. Dysfunctional adipose tissue in obesity is commonly associated with an increased recruitment of pro‐inflammatory M1 macrophages, Th1 and Th17 CD4+ and B cells, neutrophils and dendritic cells [11], all known potential targets of SARS‐CoV‐2 and contributors to the cytokine storm [6, 11, 12]. In addition, adipocytes actively participate in shaping the inflammatory milieu through the secretion of immunoregulatory cytokines (TNF‐α, IL‐1β, IL‐6 and IFN‐γ), chemokines (MCP‐1, IL‐8, CCL2 and CCL5) and alarmins (HMGB1, tenascin C and calprotectin) [11, 38]. The SARS‐CoV‐2 spike glycoprotein S1 activates Toll‐like receptor 4 (TLR4) in macrophages, leading to exaggerated cytokine production via NF‐κB‐dependent pathways [49]. Consistent with these findings, our study confirms that S1 exposure promotes macrophage polarisation toward a pro‐inflammatory M1 phenotype, accompanied by increased secretion of the alarmin HMGB1. In adipocytes, viral S1 can trigger NLRP3‐induced pyroptosis, apoptosis and MLKL‐related necroptosis, also known as PANoptosis or inflammatory cell death [28, 50]. Here, we demonstrate that S1 induces a pro‐inflammatory and dysfunctional phenotype in human visceral adipocytes, as evidenced by the reduced anti‐inflammatory adipokine adiponectin and increased HMGB1 expression. In agreement with this, Reiterer and colleagues [7] reported that SARS‐CoV‐2 infection of adipose tissue in a hamster model resulted in decreased adiponectin levels, supporting a link between adipose infectivity and dysfunction. Given that obesity is already associated with reduced adiponectin levels [51] and increased apoptosis [33], NLRP3 inflammasome assembly [34] and HMGB1 secretion [39] in VAT, these alterations may predispose adipose tissue to exacerbated S1‐driven dysfunction (Figure 6).

Given that FNDC4 and FNDC5 induce profound changes in adipose tissue mass, thermogenesis and inflammation [15, 16, 17, 18, 19, 20, 21, 22], we hypothesised that these adipo‐myokines might protect against S1‐induced adipose tissue dysfunction targeting both adipocytes and macrophages. In a previous study, our group demonstrated that FNDC4 and FNDC5 attenuate the expression of SARS‐CoV‐2 host cell receptors (ACE2, CD147, DPP4 and NRP1) and prevent S1‐induced initiation of PANoptosis in human visceral adipocytes [28]. Moreover, FNDC5 treatment attenuates SARS‐CoV‐2 entry [30] and reverses gene expression changes associated with viral infection in human subcutaneous adipocytes [29], including the downregulation of TRIB3 and the normalisation of genes involved in viral processing and entry (TLR3, HAT1, HDAC2, KDM5B, SIRT1, RAB1A, FURIN and ADAM10). In the present study, we found that FNDC4 and FNDC5 are expressed in adipose tissue macrophage populations that also express the SARS‐CoV‐2 receptor ACE2. Notably, the expression of FNDC4 was positively correlated with SARS‐CoV‐2 entry receptors (ACE2, CD147, DPP4, NRP1) in VAT, but these associations should not be interpreted as evidence of a direct causal relationship. Importantly, FNDC4 is highly expressed in macrophages [20], and its increased expression in VAT might be closely linked to macrophage infiltration, which is known to be elevated in people living with obesity [52]. Therefore, the positive correlations observed between FNDC4 and viral entry receptors may reflect changes in adipose tissue cellular composition, particularly increased macrophage recruitment, rather than a direct stimulatory effect of FNDC4 on the expression of these receptors.

Both adipo‐myokines have been shown to regulate macrophage host defence by promoting an anti‐inflammatory M2 phenotype in inflammatory conditions and cancer [20, 23, 53, 54, 55]. During the co‐incubation of macrophages with S1 and FNDC5, the increase in M1 markers (*IL1B* and *NOS2*) was maintained; however, the concurrent induction of anti‐inflammatory mediators such as *ARG1* and *IL10* suggests the activation of compensatory or resolution‐associated pathways that may contribute to limiting excessive inflammation. The simultaneous induction of pro‐ and anti‐inflammatory genes may appear contradictory if interpreted within the classical binary M1/M2 polarisation framework. However, increasing evidence indicates that macrophage activation in metabolic and inflammatory environments in adipose tissue is highly dynamic and cannot be fully captured by a strict dichotomous classification [56]. Instead, macrophages frequently exhibit mixed or transitional phenotypes characterised by the concurrent expression of both pro‐ and anti‐inflammatory markers. In line with this observation, adipocyte‐conditioned media from people with obesity amplified S1‐driven pro‐inflammatory macrophage activation. Under these conditions, both FNDC4 and FNDC5 significantly increased the expression of the anti‐inflammatory markers ARG1 and IL10 and reduced the secretion of the alarmin HMGB1, indicating a net anti‐inflammatory shift in macrophage activation consistent with previous studies [54, 55]. These observations are further supported by recent evidence demonstrating that FNDC5/irisin exerts anti‐inflammatory and immunoregulatory effects in adipose tissue through modulation of IL‐33 signalling and regulatory T‐cell responses [57]. Supporting these anti‐inflammatory actions, FNDC4 and FNDC5 also reduced S1‐induced HMGB1 expression in adipocytes, whereas gene silencing of either adipo‐myokine increased HMGB1 levels in differentiated adipocytes. Collectively, these findings support that FNDC4 and FNDC5 modulate the immune crosstalk between adipocytes and macrophages in the context of SARS‐CoV‐2 exposure, thereby mitigating adipose tissue inflammation and dysfunction. Patients with obesity might be at higher risk of SARS‐CoV‐2 infection and S1‐induced adipose tissue dysfunction due to their low circulating FNDC4 and FNDC5 levels (Figure 6).

Our study has several limitations. The measurement of FNDC5 using commercial ELISA kits remains a subject of ongoing controversy due to concerns regarding antibody specificity, including binding to non‐specific serum proteins and substantial variability in reported concentrations ranging from pg/mL to μg/mL [58]. Although the circulating FNDC5/irisin concentrations observed in our cohort were comparable to those reported in studies employing mass spectrometry with heavy stable isotope‐labelled control peptides as internal standards (~3.6 ng/mL), the gold standard method for irisin measurement [59], we acknowledge that FNDC5 concentrations in our samples were determined using an ELISA‐based method that has not been independently validated against mass spectrometry in this specific cohort. Therefore, absolute concentration values should be interpreted with caution. Nevertheless, the observed group differences and associations with clinical and inflammatory parameters are consistent with previous studies using similar methodologies [60, 61, 62, 63, 64], supporting the potential biological relevance of the findings. In addition, larger cohorts will be required to enhance statistical power, and to more definitively establish FNDC4 and FNDC5 contributions to the susceptibility to severe COVID‐19 outcomes in people with severe obesity, potentially accounting for differences in physical activity levels with more accurate methods (e.g., accelerometry). A further limitation of this study is the use of recombinant SARS‐CoV‐2 spike S1 protein rather than live SARS‐CoV‐2, pseudovirus or in vivo infection models. Although the use of recombinant SARS‐CoV‐2 spike protein subunit 1 (S1) is sufficient to activate in vitro inflammatory pathways in human endothelial [65] and vascular [66] cells, adipocytes [28], monocytes [38], and macrophages [67], all of which are key cellular components of adipose tissue, this approach does not recapitulate key aspects of viral infection, including viral entry dynamics, replication, assembly and the coordinated innate and adaptive immune responses that occur in vivo. Therefore, our findings should be interpreted as reflecting S1‐mediated inflammatory signalling rather than the full spectrum of host‐virus interactions. While recombinant S1 protein represents a widely used and informative tool to investigate specific spike‐driven cellular responses in a controlled experimental setting, validation of these observations in more physiologically relevant models, such as viral infection systems or in vivo studies, will be important to confirm the translational relevance of the identified mechanisms. Furthermore, a major strength of this study lies in the careful selection of participants to minimise confounding factors, together with comprehensive clinical, biochemical and metabolic characterisation, including detailed anthropometric measurements and body composition assessment, enabling robust and well‐controlled analyses.

## 5 Conclusions

In summary, we provide evidence that FNDC4 and FNDC5 participate in the regulation of adipocyte‐macrophage immune interactions during SARS‐CoV‐2 exposure. In obesity, reduced levels of these adipo‐myokines, together with increased expression of SARS‐CoV‐2 receptors in visceral fat, may predispose this depot to heightened S1‐induced inflammatory responses.

## Author Contributions

A.R. conceived and designed the study. G.N., J.H.‐C., V.C., S.B., M.M., V.V., R.M., C.S., J.G.‐A., J.E., G.F. and A.R. collected and analysed data. G.N., J.H.‐C. and A.R. drafted the manuscript. S.B., V.C., J.G.‐A. and G.F. revised the manuscript critically for important intellectual content. All the authors participated in final approval of the version to be published. A.R. and G.F. secured funding. A.R. and G.F. are the guarantors of this work and contributed equally to it, had full access to all the data, and take full responsibility for the integrity of data and the accuracy of data analysis.

## Funding

G.N. is the recipient of a predoctoral grant from the Department of University, Innovation and Digital Transformation of the Gobierno de Navarra (exp. 0011‐0537‐2023‐000102). This work was supported by Fondo de Investigación Sanitaria‐FEDER (PI22/00223, PI22/00745 and PI25/00528) and CIBEROBN from the Spanish Instituto de Salud Carlos III and the Department of Health of the Gobierno de Navarra (exp. 0011‐3638‐2020‐000002 and GN2025/52).

## Conflicts of Interest

Gema Frühbeck received payment of honoraria from Lilly, Novo Nordisk, Regeneron, AstraZeneca and Boehringer Ingelheim as a member of advisory boards, and payment of honoraria for lectures as a member of the OPEN Spain Initiative. The other authors declared no conflicts of interest.

## Supporting information


**Table S1:** Sequences of primers and TaqMan probes.
**Figure S1:** Efficiency of gene silencing of *FNDC4* and *FNDC5* genes in human visceral adipocytes. *FNDC4* (A) and *FNDC5* (B) mRNA levels in human omental differentiated adipocytes after knockdown of *FNDC4* and *FNDC5* gene expression, respectively, with esiRNA for 24 h. Values are the mean ± SEM (*n* = 4–7 per group). Differences between groups were analysed by Student's *t*‐test *p < 0.05; ***p < 0.001 versus control esiRNA cells.

## Data Availability

The data contained within the article are available on request from the corresponding author.
