# Supplementary material for: FNDC4 and FNDC5 Attenuate SARS‐CoV‐2 S1‐Induced Inflammatory Responses in Human Adipose Tissue
Source: Eur J Clin Invest. 2026 Apr 28;56:e70215. doi: 10.1111/eci.70215 (PMC13123202; doi:10.1111/eci.70215)
Supplement: Supplementary file 1 — Table S1: Sequences of primers and TaqMan probes. Figure S1: Efficiency of gene silencing of FNDC4 and FNDC5 genes in human visceral adipocytes. FNDC4 (A) and FNDC5 (B) mRNA levels in human omental differentiated adipocytes after knockdown of FNDC4 and FNDC5 gene expression, respectively, with esiRNA for 24 h. Values are the mean ± SEM (n = 4–7 per group). Differences between groups were analysed by Student's t‐test *p < 0.05; ***p < 0.001 versus control esiRNA cells. [file ECI-56-e70215-s001.docx]

**Supplemental Table 1. Sequences of primers and TaqMan^®^ probes.**

| Gene  (GenBank  accession number) | Oligonucleotide sequence (5’-3’) | Nucleotides |
| --- | --- | --- |
| *ACE2*  (NM_001371415.1)  Forward  Reverse  Probe | CCCTTTGGACAGAAACCAAACA  AAGGAGGCCGAGAAGTTCTTTG  FAM-CAGGCCTGGGATGCACAGAGAATAT-TAMRA | 899-920  974-995  947-971 |
| *CD147*  (NM_001728.4)  Forward  Reverse  Probe | CACTGACTGGGCCTGGTACAA  GAGAACCTGAACATGGAGGCC  FAM-CTGAGGACAAGGCCCTCATGAACGG-TAMRA | 789-809  901-921  821-845 |
| *DPP4*  (NM_001379604.1)  Forward  Reverse  Probe | GGTCTCCAAACGGCACTTTTT  CCATATCCAAAGGCAGGAGCT  FAM-CCCAATTTAACGACACAGAAGTCCCAC-TAMRA | 877-898  994-1014  908-934 |
| *FNDC4*  (NM_022823.2)  Forward  Reverse  Probe | TGGTCATCATTGTGGTGGTGTT  AGAGTCCTCAGGGAAGGCCA  FAM-CATGTGGGCTGCTGTAATTGGGCTGTT-TAMRA | 915-936  1041-1060  940-966 |
| *FNDC5*  (NM_001171940.1)  Forward  Reverse  Probe | TGAGGTTGTCATCGGATTTGC  AACACCACCACCCGCTCAT  FAM-CTCCCAGCAGAAGAAGGATGTGCGG-TAMRA | 244-264  317-335  268-292 |
| *FURIN*  (NM_001289823.2)  Forward  Reverse  Probe | GACTGACTTGCGGCAGAAGTG  ATAAGAACCTCACATGGCGGG  FAM-TTAGCAGCCGGCATCATTGCTCTCA-TAMRA | 1284-1304  1379-1399  1342-1366 |
| *NRP1*  (NM_003873.7)  Forward  Reverse  Probe | ACAATCACGTGCAGCTCAAGTG  ATTCCCAAGCTGACGAAAATCA  FAM-TCAGGATCACACAGGAGATGGCAACT-TAMRA | 2282-2303  2366-2387  2334-2359 |

*ACE2,* angiotensin I converting enzyme 2; *CD147*, basigin (Ok blood group) (BSG); *DPP4*, dipeptidyl peptidase 4; *FNDC4*, fibronectin type III domain containing 4; *FNDC5*, fibronectin type III domain containing 5; *FURIN*, furin, paired basic amino acid cleaving enzyme; *NRP1*, neuropilin 1.


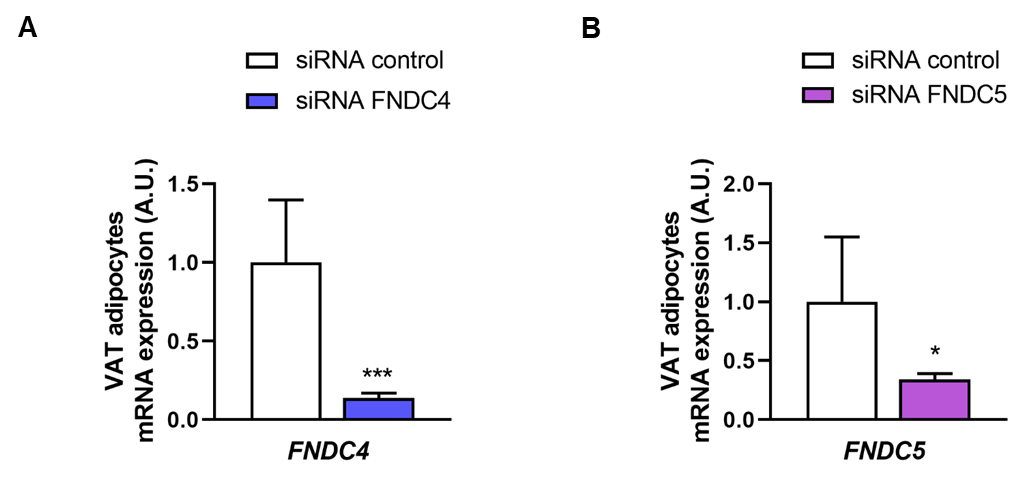


**Supplemental Fig. 1. Efficiency of gene silencing of *FNDC4* and *FNDC5* genes in human visceral adipocytes**. *FNDC4* (A) and *FNDC5* (B) mRNA levels in human omental differentiated adipocytes after knockdown of *FNDC4* and *FNDC5* gene expression, respectively, with esiRNA for 24 h. Values are the mean ± SEM (n=4-7 per group). Differences between groups were analyzed by Student’s *t* test **P*<0.05; ****P*<0.001 *vs* control esiRNA cells.
